# Bilateral ciliary body cysts mimicking peripheral bullous retinal detachment: Diagnosis by ultrasound biomicroscopy and glaucomatous implications

**DOI:** 10.1016/j.ajoc.2026.102647

**Published:** 2026-08-25

**Authors:** Sami Bounetta, Victor Vermot-Desroches, Etienne Weinzaepfel, Ahmed Rahmi, Thibaud Mathis, Laurent Kodjikian, Philippe Denis

**Affiliations:** a Service d'Ophtalmologie, Hôpital Universitaire de la Croix-Rousse, Hospices Civils de Lyon, 103, Grande Rue de la Croix-Rousse, Lyon Cedex 04, 69317, France; b Centre Ophtalmologie Laser Vision, Bourgoin-Jallieu, 38300, France; c Laboratoire MATEIS, UMR-CNRS 5510, INSA, Université Lyon 1, Villeurbanne, 69100, France

## 1 Case report

A 64-year-old man with no significant medical history was referred for bilateral peripheral bullous lesions detected on fundus examination. The lesions were located in the extreme inferotemporal periphery of both eyes, appearing smooth, dome-shaped, and highly elevated, without associated retinal breaks or lattice degeneration. Fundus findings remained stable at 72-h follow-up, raising suspicion for peripheral retinoschisis versus ora dialysis, and prompting referral to a tertiary center.

**Fig. 1:**
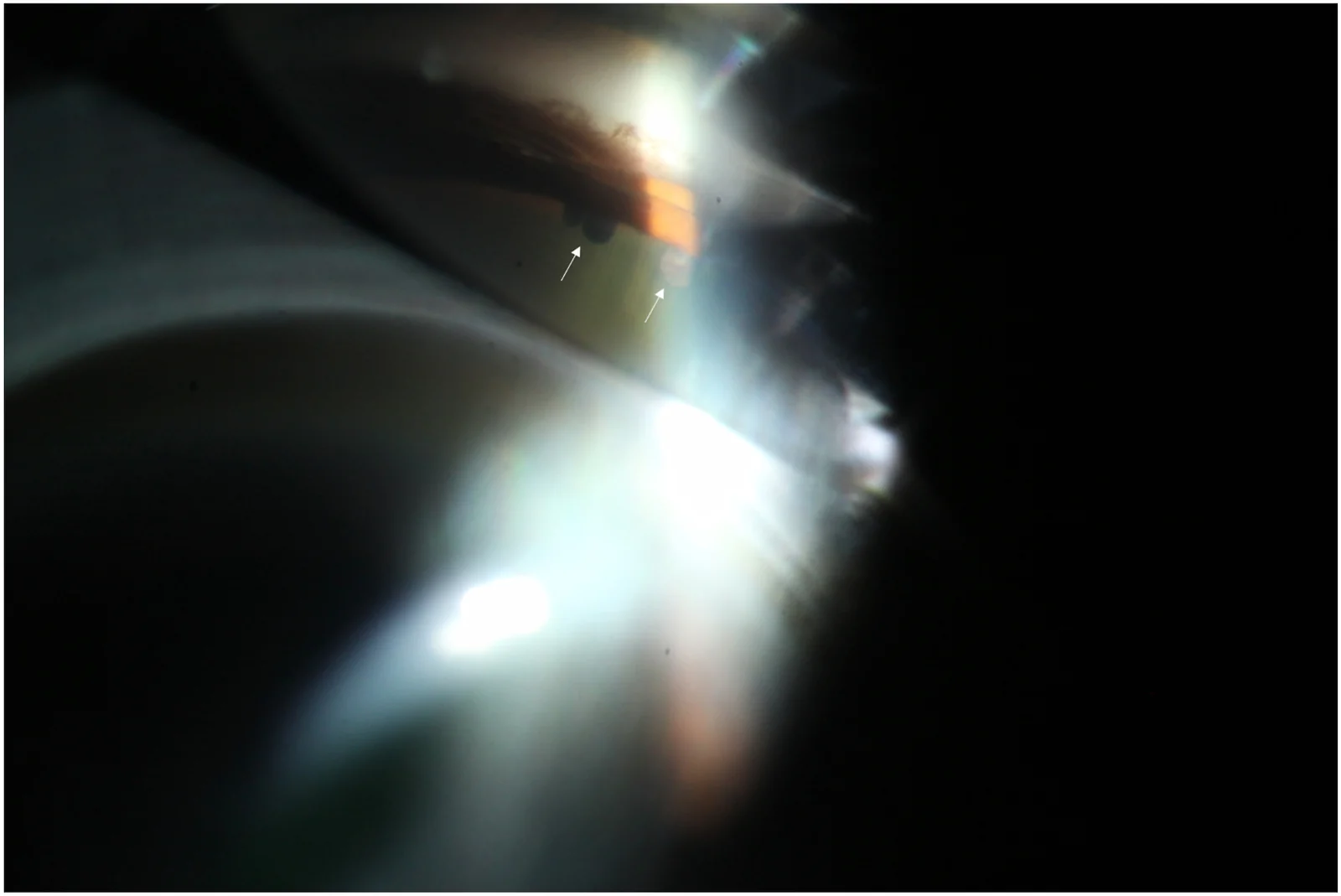
Gonioscopy (3-mirror lens) showing focal angle narrowing corresponding to cyst location.

At presentation, best-corrected visual acuity was 20/20 in both eyes. Slit-lamp examination was unremarkable, with a quiet anterior chamber, no cells or flare, normal iris architecture, clear crystalline lenses, and a clear vitreous. Intraocular pressure (IOP) was within normal limits in both eyes on repeated measurements. Gonioscopy using a three-mirror lens demonstrated focal angle narrowing in the corresponding quadrant (Fig. 1). Indentation gonioscopy failed to widen the angle, consistent with localized synechial rather than appositional angle closure.

**Fig. 2:**
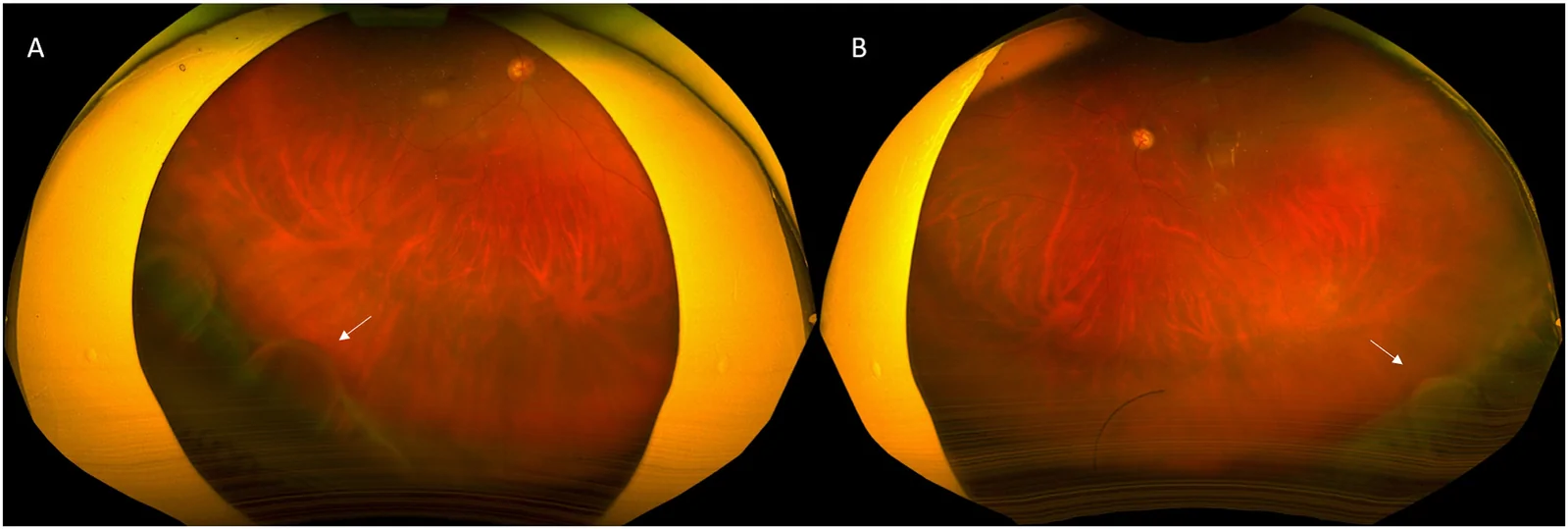
Ultra-widefield fundus imaging showing bilateral inferotemporal bullous peripheral elevations. (A) Right eye. (B) Left eye.

**Fig. 3:**
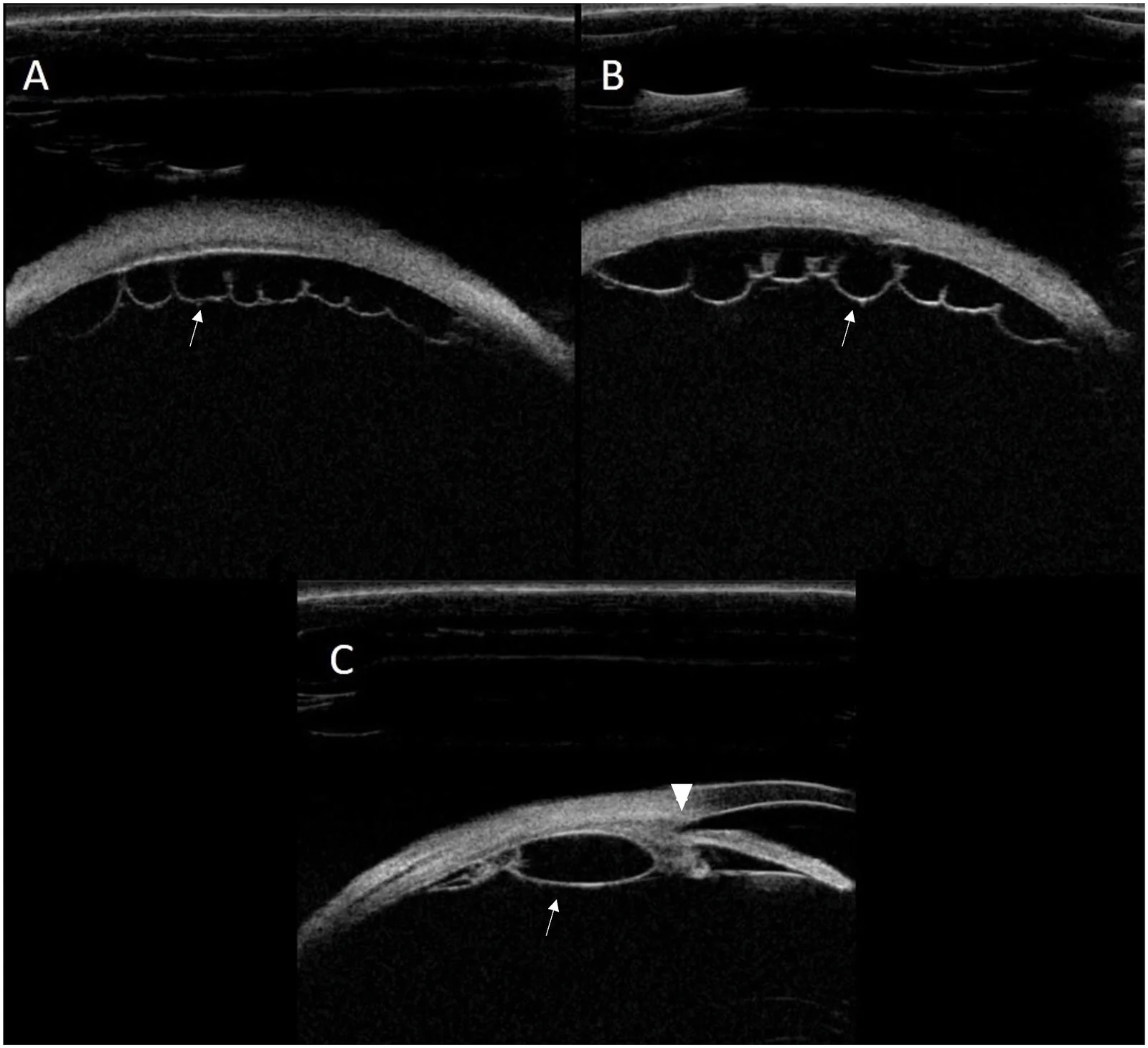
Ultrasound biomicroscopy demonstrating multiple ciliary body cysts (arrows) in the inferotemporal quadrant of both eyes. (A) Right eye. (B) Left eye. (C) Detailed view showing a large ciliary body cyst (arrow) associated with focal iridocorneal angle narrowing (arrowhead).

Dilated fundus examination confirmed anteriorly located cystic-appearing elevations in the inferotemporal periphery of both eyes (Fig. 2). Fluorescein and indocyanine green angiography were unremarkable. Optic disc examination showed a cup-to-disc ratio of 0.7 bilaterally. Retinal nerve fiber layer (RNFL) analysis revealed asymmetric thinning: 81 μm in the right eye (temporal and superotemporal sectors) and 53 μm in the left eye (predominantly temporal). Standard automated perimetry revealed an early glaucomatous defect in the left eye, consistent with the structural RNFL loss. Anterior-segment OCT failed to visualize the ciliary body adequately. Ultrasound biomicroscopy (UBM) revealed multiple bilateral ciliary body cysts in the inferotemporal quadrant of both eyes (Fig. 3), explaining the apparent fundus elevation. Findings remained stable over 1 year, with normal IOP and unchanged cysts. Owing to the structural and functional glaucomatous abnormalities, prostaglandin monotherapy was initiated.

## 2 Discussion

Ciliary body cysts are usually benign and incidental lesions of the nonpigmented ciliary epithelium.^1^ However, when multiple and anteriorly located, they may induce peripheral retinal elevation and mimic peripheral retinoschisis, pars plana cysts, ciliochoroidal detachment, peripheral exudative retinal lesions, or retinal detachment.^2^

Standard B-scan ultrasonography may fail to detect small or anteriorly located cysts. Anterior-segment OCT may also inadequately visualize peripheral ciliary body lesions, whereas UBM remains the gold standard for imaging the ciliary body, providing high-resolution visualization.

Focal angle narrowing was consistent with a plateau iris-like configuration likely resulting from anterior displacement of the iris root by multiple ciliary body cysts.^3^ Although RNFL thinning and a corresponding visual field defect were present, IOP remained normal throughout follow-up. Therefore, a causal relationship cannot be established, and normal-tension glaucoma remains a plausible alternative diagnosis. Laser peripheral iridotomy would not be expected to relieve angle narrowing caused by the mechanical effect of the cysts. Although ciliary body cysts have occasionally been associated with systemic disorders such as paraproteinemia, no clinical features suggested an underlying systemic disease in our patient. These findings underscore the value of UBM in distinguishing ciliary body cysts from other causes of peripheral retinal elevation, including the rare underlying tissue lesions with potential malignant features.

## 3 Conclusion

Bilateral ciliary body cysts may closely mimic peripheral bullous retinal pathology and lead to diagnostic uncertainty. UBM is essential for accurate diagnosis in such cases. Although focal angle narrowing and glaucomatous abnormalities coexisted, causality cannot be inferred.

This case highlights the importance of UBM and gonioscopy in the evaluation of anterior peripheral fundus lesions, particularly in the setting of focal angle closure, and supports long-term structural and functional glaucoma monitoring.

## CRediT authorship contribution statement

**Sami Bounetta:** Writing – review & editing, Writing – original draft, Supervision, Methodology, Conceptualization. **Victor Vermot-Desroches:** Writing – review & editing, Validation, Formal analysis. **Etienne Weinzaepfel:** Methodology, Formal analysis, Conceptualization. **Ahmed Rahmi:** Software, Resources, Data curation. **Thibaud Mathis:** Writing – review & editing, Project administration. **Laurent Kodjikian:** Writing – review & editing, Validation. **Philippe Denis:** Writing – review & editing, Writing – original draft, Validation, Conceptualization.

## Patient consent

Written informed consent was provided by the patient for the use of identifiable data and photographs.

## Acknowledgements and disclosures

No funding or grant support.

## Authorship

All authors attest that they meet the current ICMJE criteria for Authorship.

## Financial Support

None.

## Declaration of competing interests

The authors declare that they have no known competing financial interests or personal relationships that could have appeared to influence the work reported in this paper.
